# The complete mitochondrial genome of *Baikalospongia intermedia* (Lubomirskiidae): description and phylogenetic analysis

**DOI:** 10.1080/23802359.2016.1172273

**Published:** 2016-08-21

**Authors:** Olga Maikova, Dmitry Sherbakov, Sergei Belikov

**Affiliations:** Limnological Institute SB RAS, Irkutsk, 664033, Russian Federation

**Keywords:** Lake Baikal, mitochondrial genome, sponges

## Abstract

The complete mitochondrial genome of the Lake Baikal sponge *Baikalospongia intermedia* was sequenced. The circular mitochondrial genome is 28,327 bp in length and includes 14 protein-coding genes, 2 ribosomal RNA genes and 25 transfer RNA genes. Bayesian comparative analysis of molecular evolution rates was found no acceleration of the mtDNA evolution of *B. intermedia*. This species clustered with other species of the genus *Baikalospongia* on the Bayesian tree.

Sponges of Lake Baikal (*Lubomirskiidae*) are an endemic group of freshwater sponges (*Demospongiae, Haplosclerida*) that originated from the cosmopolitan family *Spongillidae* (Itskovich et al. 2008) during last *ca*. 10 MYA according to the molecular phylogeny (Maikova et al. 2015). Phylogenetic relationships within the family *Lubomirskiidae* are still poorly understood (Efremova 2001; Itskovich et al. 2006, 2008). Earlier, in order to reveal phylogenetic relationships, sequences of six mitochondrial genomes of Baikal sponges were determined (Lavrov 2010; Lavrov et al. 2012; Maikova et al. 2015). Here, we described a new mtDNA of *Baikalospongia intermedia*, which will be a useful instrument for future phylogenetic analyses of Baikal endemic sponges.

*B. intermedia* is one of the most abundant species in Lake Baikal, which is distributed widely in the lake at the depth range of 3 to 40 m (Efremova 2001). A specimen was collected from a depth of 20 m near the village of Bolshiye Koty (51°54" 16.66" N, 105°5′ 28.36" E) in August 2008 and kept under number BS121 in the sponge collection at Laboratory of Analytical and Bioorganic Chemistry of Limnological Institute (Irkutsk, Russia).

The species was identified from spicules and skeleton of the sponge. Spicule and skeleton preparations were performed as previously described (Efremova 2001). Total DNA was extracted from tissue by a modified phenol–chloroform method (Maniatis et al. 1984). Genome was amplified and sequenced as previously described (Maikova et. al. 2015). The genome was assembled using MAFFT v 6.882 (Katoh & Toh 2008). In mitochondrial DNA sequence of *B. intermedia*, it was problematic to sequence a short fragment within the gene for small subunit ribosomal RNA (*rns*) and a section of the intergenic region (IGR) between *trnQ* and *trnN* genes, including *trnW* gene, because of probable additional hairpin structures. Expected total length of problematic regions is 1186 bp based on the alignment to the closely related mitochondrial genome of *L. baicalensis*. Annotated mitochondrial genome sequence of *B. intermedia* is available in NCBI (GenBank accession number KU324767).

The mtDNA of *B. intermedia* is 28,327 bp and contains 2 rRNA genes, 25 tRNA genes and 14 protein-coding genes. Total length of intergenic regions is 9136 bp, which constitutes 32% of the genome. The A + T base composition of the genome is 59.05%, the A + T content of genes ranging from 48.86% to 68.94% (Table 1).

**Table 1.** The genomic organization of mitochondrial genes of *Baikalospongia intermedia*.

Gene	Length, bp	A+T, %	Anti/start codon	Stop codon
*rnl*	2997	58.43	AAG	CAT
*Cox2*	777	67.82	ATG	TAA
*trnK*	73	58.90	TTT	–
*Atp8*	255	64.31	ATG	TAG
*Atp6*	735	68.84	ATG	TAA
*trnR*	74	60.81	TCT	–
*Cox3*	789	66.41	ATG	TAA
*trnQ*	73	56.16	TTG	–
*trnW*^a^	71	57.75	TCA	–
*trnN*	72	66.67	GTT	–
*trnL*	74	56.76	TAG	–
*Cob*	1149	67.28	ATG	TAG
*trnT*	74	58.11	TGT	–
*Atp9*	237	63.29	ATG	TAA
*trnS*	88	48.86	GCT	–
*trnP*	73	53.42	TGG	–
*Nad4*	1452	68.94	ATG	TAA
*trnH*	74	51.35	GTG	–
*trnE*	72	55.56	TTC	–
*Nad6*	588	65.82	GTG	TAA
*Nad3*	357	70.31	ATG	TAA
*trnR*	71	66.20	TCG	–
*Nad4L*	300	72.33	ATG	TAG
*Cox1*	1581	65.72	ATG	TAA
*trnS*	86	52.33	TGA	–
*trnD*	73	61.64	GTC	–
*trnC*	73	63.01	GCA	–
*Nad1*	975	67.79	ATG	TAA
*trnL*	76	57.89	TAA	–
*trnI*	74	56.76	GAT	–
*trnY*	84	51.19	GTA	–
*trnI*	73	61.64	CAT	–
*trnM*	72	56.94	CAT	–
*Nad2*	1488	65.52	TTG	TAA
*Nad5*	1881	68.79	ATG	CCA
*trnA*	74	51.35	TGC	–
*trnM*	71	67.61	CAT	–
*trnF*	74	59.46	GAA	–
*rns*	1,767	57.19	ATT	AAT
*trnG*	72	62.50	TCC	–
*trnV*	73	56.16	TAC	–

a The sequence was analyzed from closely related mitochondrial genome of *L. baicalensis*.

The genome contains tRNA genes for all amino acids. However, the anticodons encoded match only 39% of all codons in the genome. For example, tRNA^Ile^ with codon AUC matching GAU is only 8.7% of all codons coding this amino acid. Supposedly, at least some synonymic mutations may be nonneutral in case if codon composition affects the translation rate of the corresponding gene (Zhou et al. 2013).

Bayesian comparative analysis (Ronquist & Huelsenbeck 2003) found no significant difference between mitochondrial DNA substitution rate of *B. intermedia* and other Baikal sponges (95% HPD interval <1, variance >1). *B. intermedia* clustered on the phylogenetic tree with other species of the genus *Baikalospongia* (Figure 1).

**Figure 1.**
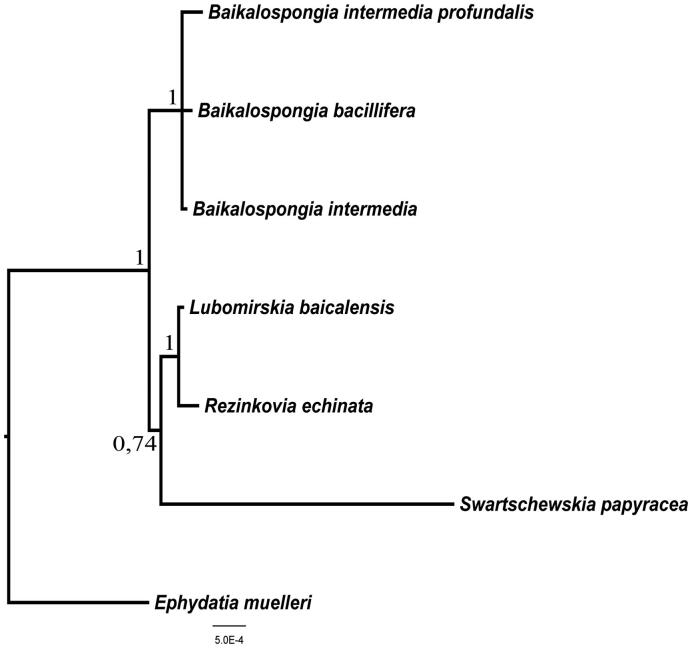
Bayesian tree inferred from coding sequences of all 14 mitochondrial genes of six species from *Lubomirskiidae* (*R. echinata* (JQ302309), *S. papyracea* (JQ302308), *B. intermedia profundalis* (JQ302310), *L. baicalensis* (GU385217), *B. bacillifera* (KJ192328), *B. intermedia* (KU324767)) and one species from *Spongillidae* (*E. muelleri* (NC_010202)) as out group. For Bayesian analyses, the Markov chain Monte Carlo search was run twice (default parameter) on four chains for 5,000,000 generations/trees were sampled every 1000th cycle after the first 10,000 burn-in cycles. Values above and to the left of nodes are Bayesian posterior probabilities.
